# Supplementary material for: Polyketide synthase gene clusters in the ericoid mycorrhizal fungus Oidiodendron maius: genome-wide prediction, expression profiling, and functional characterization of knockout mutants
Source: Front Plant Sci. 2026 Sep 16;17:1924430. doi: 10.3389/fpls.2026.1924430 (PMC13623903; doi:10.3389/fpls.2026.1924430)
Supplement: Supplementary file 1 [file SupplementaryFile1.zip › Supplementary Materials/Supplementary Folder 1.zip!/Oidma1_AssemblyScaffolds_Repeatmasked/knownclusterblast/cluster11/jgi.p_Oidma1_39552_mibig_hits.html]

| MIBiG Protein | Description | MIBiG Cluster | MiBiG Product | % ID | % Coverage | BLAST Score | E-value |
| --- | --- | --- | --- | --- | --- | --- | --- |
| AAC49205.1 | putative\_ketoreductase | BGC0000152 | Sterigmatocystin\_biosynthetic\_gene\_cluster | 46 | 98.1343283582 | 234 | 1.2e-61 |
| ACI31322.1 | AflM | BGC0000011 | Aflatoxin/sterigmatocystin\_biosynthetic\_gene\_cluster | 48 | 98.8805970149 | 233 | 2.7e-61 |
| AAS90099.1 | Ver-1 | BGC0000006 | Aflatoxin\_biosynthetic\_gene\_cluster | 49 | 95.5223880597 | 233 | 3.6e-61 |
| AAS90008.1 | Ver-1 | BGC0000007 | Aflatoxin\_biosynthetic\_gene\_cluster | 49 | 95.5223880597 | 233 | 3.6e-61 |
| AAS90030.1 | Ver-1 | BGC0000008 | Aflatoxin\_biosynthetic\_gene\_cluster | 49 | 95.5223880597 | 233 | 3.6e-61 |
| AAS90076.1 | Ver-1 | BGC0000010 | Aflatoxin\_biosynthetic\_gene\_cluster | 49 | 95.5223880597 | 233 | 3.6e-61 |
| CBF90105.1 | oxidoreductase,\_putative\_(Eurofung) | BGC0001271 | Emericellin\_biosynthetic\_gene\_cluster | 50 | 95.5223880597 | 233 | 3.6e-61 |
| AAS90056.1 | Ver-1 | BGC0000009 | Aflatoxin\_biosynthetic\_gene\_cluster | 49 | 95.5223880597 | 232 | 4.7e-61 |
| BAE71323.1 | reductase/dehydrogenase | BGC0000004 | Aflatoxin\_biosynthetic\_gene\_cluster | 49 | 95.5223880597 | 232 | 8e-61 |
| ABZ01830.1 | ESC\_reductase | BGC0001259 | Elsinochrome\_A\_biosynthetic\_gene\_cluster | 44 | 99.2537313433 | 224 | 1.7e-58 |
| AFO85456.1 | reductase/oxidase | BGC0000391 | Methylpendolmycin/pendolmycin\_biosynthetic\_gene\_cluster | 41 | 96.6417910448 | 180 | 2.8e-45 |
| AGN74904.1 | ketoreductase | BGC0000459 | Griseoviridin\_/\_viridogrisein\_biosynthetic\_gene\_cluster | 40 | 97.3880597015 | 160 | 3e-39 |
| ACN38371.1 | short-chain\_dehydrogenase/reductase | BGC0000714 | Sisomicin\_biosynthetic\_gene\_cluster | 39 | 94.0298507463 | 157 | 1.9e-38 |
| CAG15000.1 | short\_chain\_dehydrogenase | BGC0000441 | Teicoplanin\_biosynthetic\_gene\_cluster | 37 | 93.6567164179 | 152 | 6.2e-37 |
| CAE53341.1 | putative\_short\_chain\_dehydrogenase | BGC0000440 | Teicoplanin\_biosynthetic\_gene\_cluster | 37 | 93.6567164179 | 152 | 6.2e-37 |
| AAT12286.1 | LtxD | BGC0000384 | Lyngbyatoxin\_biosynthetic\_gene\_cluster | 36 | 95.1492537313 | 151 | 1.4e-36 |
| AEF33074.1 | 3-oxoacyl-[acyl-carrier\_protein]\_reductase | BGC0001039 | Pyridomycin\_biosynthetic\_gene\_cluster | 38 | 95.1492537313 | 150 | 2.3e-36 |
| BAF85836.1 | C5-ketoreductase | BGC0000109 | Nemadectin\_biosynthetic\_gene\_cluster | 37 | 100.746268657 | 150 | 3.1e-36 |
| AKA54637.1 | 3-oxoacyl-ACP\_reductase | BGC0001216 | Splenocin\_biosynthetic\_gene\_cluster | 36 | 97.3880597015 | 147 | 2e-35 |
| CAC37883.1 | putative\_3-oxoacyl-[acyl-carrier\_protein]\_reductase | BGC0000038 | Coelimycin\_biosynthetic\_gene\_cluster | 35 | 97.7611940299 | 146 | 3.4e-35 |
| WP\_010639249.1 | short-chain\_dehydrogenase | BGC0000958 | Antimycin\_biosynthetic\_gene\_cluster | 36 | 97.3880597015 | 145 | 7.5e-35 |
| ABX71123.1 | Lct40 | BGC0000238 | Lactonamycin\_biosynthetic\_gene\_cluster | 35 | 95.5223880597 | 145 | 9.8e-35 |
| AAQ84160.1 | ChcA | BGC0000123 | Phoslactomycin\_B\_biosynthetic\_gene\_cluster | 36 | 93.6567164179 | 144 | 1.7e-34 |
| AAK57530.1 | PgaM | BGC0000262 | Rabelomycin\_biosynthetic\_gene\_cluster | 33 | 97.7611940299 | 143 | 2.9e-34 |
| AMK51276.1 | Hex19 | BGC0001376 | Hexaricin\_biosynthetic\_gene\_cluster | 35 | 95.5223880597 | 143 | 3.7e-34 |
| AAX57196.2 | auricin\_polyketide\_oxygenase-reductase | BGC0000201 | Auricin\_biosynthetic\_gene\_cluster | 32 | 97.7611940299 | 141 | 1.1e-33 |
| ACP19359.1 | SaqN | BGC0000267 | Saquayamycin\_Z\_/\_galtamycin\_B\_biosynthetic\_gene\_cluster | 36 | 97.7611940299 | 141 | 1.4e-33 |
| CAH10112.1 | putative\_oxidase-reducatse | BGC0000268 | Sch47554\_/\_Sch47555\_biosynthetic\_gene\_cluster | 33 | 97.7611940299 | 136 | 4.6e-32 |
| BAJ52708.1 | putative\_3-ketoacyl-ACP\_reductase | BGC0000222 | FD-594\_biosynthetic\_gene\_cluster | 34 | 95.5223880597 | 136 | 4.6e-32 |
| AHH25583.1 | 1-cyclohexenylcarbonyl\_CoA\_reductase | BGC0000957 | Ansatrienin\_(mycotrienin)\_biosynthetic\_gene\_cluster | 34 | 93.6567164179 | 135 | 7.8e-32 |
| CAI94720.1 | putative\_oxyacyl-(acyl\_carrier\_protein)\_reductase | BGC0000141 | Rubradirin\_biosynthetic\_gene\_cluster | 38 | 94.4029850746 | 135 | 1e-31 |
| WP\_030267262.1 | 3-oxoacyl-ACP\_reductase | BGC0001308\_c2 | Simocyclinone\_D9\_/\_simocyclinone\_D10\_/\_simocyclinone\_D11 | 37 | 95.1492537313 | 134 | 1.3e-31 |
| AGO50615.1 | oxygenase-reductase | BGC0000229 | Grincamycin\_biosynthetic\_gene\_cluster | 34 | 97.7611940299 | 134 | 2.3e-31 |
| ADI71448.1 | putative\_oxygenase-reductase | BGC0000203 | BE-7585A\_biosynthetic\_gene\_cluster | 32 | 97.7611940299 | 133 | 5.1e-31 |
| AAF00206.1 | oxygenase-reductase | BGC0000276\_c2 | Urdamycin\_biosynthetic\_gene\_cluster | 32 | 97.0149253731 | 133 | 5.1e-31 |
| BAV17004.1 | putative\_oxygenase-reductase | BGC0001384 | Saprolmycin\_biosynthetic\_gene\_cluster | 33 | 97.7611940299 | 132 | 6.6e-31 |
| BAJ07846.1 | putative\_oxygenase-reductase | BGC0000232 | Hatomarubigin\_biosynthetic\_gene\_cluster | 35 | 104.850746269 | 132 | 8.6e-31 |
| WP\_030269276.1 | short-chain\_dehydrogenase | BGC0001308 | Simocyclinone\_D9\_/\_simocyclinone\_D10\_/\_simocyclinone\_D11 | 33 | 99.6268656716 | 131 | 1.5e-30 |
| AAL15586.1 | Sim8 | BGC0000270 | Simocyclinone\_biosynthetic\_gene\_cluster | 33 | 99.6268656716 | 130 | 2.5e-30 |
| AAK06790.1 | putative\_reductase\_SimA9 | BGC0001072 | Simocyclinone\_biosynthetic\_gene\_cluster | 32 | 99.6268656716 | 129 | 7.3e-30 |
| CAM58804.1 | BenL\_protein | BGC0000204 | Benastatin\_biosynthetic\_gene\_cluster | 34 | 95.8955223881 | 129 | 7.3e-30 |
| EHM27510.1 | monooxygenase\_FAD-binding | BGC0000235 | Kiamycin\_biosynthetic\_gene\_cluster | 33 | 98.5074626866 | 127 | 2.1e-29 |
| ADG86325.1 | ketoreductase | BGC0000190 | A-74528\_biosynthetic\_gene\_cluster | 33 | 95.5223880597 | 127 | 2.8e-29 |
| AAG34187.1 | SimJ1 | BGC0000835 | Simocyclinone\_biosynthetic\_gene\_cluster | 36 | 101.492537313 | 126 | 6.2e-29 |
| AAK06807.1 | putative\_3-keto-acyl-reductase\_SimD2 | BGC0001072 | Simocyclinone\_biosynthetic\_gene\_cluster | 36 | 101.492537313 | 126 | 6.2e-29 |
| AAL15603.1 | SimJ1 | BGC0000270 | Simocyclinone\_biosynthetic\_gene\_cluster | 36 | 101.492537313 | 126 | 6.2e-29 |
| ADB23395.1 | dehydrogenase | BGC0001062 | TLN-05220\_biosynthetic\_gene\_cluster | 33 | 94.776119403 | 126 | 6.2e-29 |
| CAG14976.1 | putative\_3-keto-acyl\_carrier\_protein\_reductase | BGC0000253 | Oviedomycin\_biosynthetic\_gene\_cluster | 33 | 98.8805970149 | 124 | 1.4e-28 |
| AHX24706.1 | 3-oxoacyl-ACP\_reductase | BGC0000200 | Arixanthomycin\_biosynthetic\_gene\_cluster | 31 | 94.776119403 | 124 | 1.8e-28 |
| ADE22311.1 | putative\_3-oxoacyl-ACP\_reductase | BGC0000279 | Xantholipin\_biosynthetic\_gene\_cluster | 32 | 94.776119403 | 124 | 1.8e-28 |
| ADB23387.1 | dehydrogenase | BGC0001062 | TLN-05220\_biosynthetic\_gene\_cluster | 34 | 95.8955223881 | 124 | 1.8e-28 |
| AMK51286.1 | Hex29 | BGC0001376 | Hexaricin\_biosynthetic\_gene\_cluster | 33 | 94.4029850746 | 124 | 2.3e-28 |
| ACM68692.1 | putative\_oxidoreductase | BGC0000298 | Aeruginosin\_biosynthetic\_gene\_cluster | 33 | 95.8955223881 | 124 | 2.3e-28 |
| ABM21753.1 | PdmG | BGC0000256 | Pradimicin\_biosynthetic\_gene\_cluster | 31 | 94.0298507463 | 123 | 3.1e-28 |
| ADD83006.1 | PtnO8 | BGC0001156 | Platencin\_biosynthetic\_gene\_cluster | 34 | 97.7611940299 | 122 | 6.8e-28 |
| AJI44188.1 | hypothetical\_protein | BGC0001193 | Thiolutin\_biosynthetic\_gene\_cluster | 34 | 99.2537313433 | 121 | 1.5e-27 |
| ABB52548.1 | 3-oxoacyl-(acyl-carrier-protein)-reductase | BGC0000047 | Dihydrochalcomycin\_biosynthetic\_gene\_cluster | 33 | 96.2686567164 | 121 | 1.5e-27 |
| ANC94959.1 | AlmK | BGC0001396 | Aldgamycin\_biosynthetic\_gene\_cluster | 33 | 96.2686567164 | 121 | 2e-27 |
| ACO31295.1 | PtmO8 | BGC0001140 | Platensimycin\_/\_platencin\_biosynthetic\_gene\_cluster | 34 | 97.7611940299 | 120 | 2.6e-27 |
| ABQ04140.1 | short-chain\_dehydrogenase/reductase\_SDR | BGC0000838 | Flexirubin\_biosynthetic\_gene\_cluster | 31 | 95.5223880597 | 119 | 5.8e-27 |
| ALG65299.1 | Cal37 | BGC0001297 | WS9326\_biosynthetic\_gene\_cluster | 32 | 94.0298507463 | 119 | 5.8e-27 |
| AIL50168.1 | putative\_ketoreductase | BGC0000213 | Colabomycin\_biosynthetic\_gene\_cluster | 31 | 93.6567164179 | 119 | 7.6e-27 |
| ACU71635.1 | short-chain\_dehydrogenase/reductase\_SDR | BGC0001154 | Cacibiocin\_biosynthetic\_gene\_cluster | 35 | 95.5223880597 | 117 | 2.2e-26 |
| AHL46721.1 | ketoreductase | BGC0001179 | Rishirilide\_B\_biosynthetic\_gene\_cluster | 32 | 96.2686567164 | 116 | 3.8e-26 |
| AFV52172.1 | ketoreductase | BGC0000081 | Kedarcidin\_biosynthetic\_gene\_cluster | 33 | 95.8955223881 | 116 | 3.8e-26 |
| AAQ08926.1 | putative\_3-ketoacyl-ACP\_reductase | BGC0000224 | Fredericamycin\_biosynthetic\_gene\_cluster | 31 | 95.5223880597 | 116 | 3.8e-26 |
| AAS79466.1 | putative\_post-PKS\_ketoreductase | BGC0000035 | Chalcomycin\_biosynthetic\_gene\_cluster | 32 | 96.2686567164 | 116 | 4.9e-26 |
| ABD29269.1 | 3-ketoacyl-acyl\_carrier\_protein\_reductase,\_putative | BGC0000943 | Staphylobactin\_biosynthetic\_gene\_cluster | 34 | 97.7611940299 | 116 | 6.4e-26 |
| CAM34373.1 | putative\_reductase | BGC0000242 | Lysolipin\_biosynthetic\_gene\_cluster | 32 | 97.7611940299 | 114 | 1.9e-25 |
| ANY57964.1 | MtcE\_3-ketoacyl-ACP\_reductase | BGC0001369 | Metatricycloene\_biosynthetic\_gene\_cluster | 32 | 95.8955223881 | 113 | 3.2e-25 |
| ADI58639.1 | AsuC7 | BGC0000187 | Asukamycin\_biosynthetic\_gene\_cluster | 32 | 93.2835820896 | 113 | 3.2e-25 |
| AAN65226.1 | putative\_3-oxoacyl-[ACP]-reductase | BGC0000832 | Clorobiocin\_biosynthetic\_gene\_cluster | 35 | 95.1492537313 | 113 | 5.4e-25 |
| AAA65204.1 | daunorubicin-doxorubicin\_polyketide\_synthase | BGC0000218 | Doxorubicin\_biosynthetic\_gene\_cluster | 33 | 95.8955223881 | 112 | 9.2e-25 |
| ALV82450.1 | putative\_3-oxoacyl-ACP\_reductase | BGC0001382 | Svaricin\_biosynthetic\_gene\_cluster | 30 | 95.5223880597 | 111 | 1.6e-24 |
| BAV17002.1 | putative\_ketoreductase | BGC0001384 | Saprolmycin\_biosynthetic\_gene\_cluster | 31 | 95.8955223881 | 111 | 1.6e-24 |
| AAU93785.1 | putative\_dehydrogenase/reductase | BGC0000054 | Erythromycin\_biosynthetic\_gene\_cluster | 30 | 100.0 | 111 | 1.6e-24 |
| CAK50780.1 | ketoreductase | BGC0000247 | Mithramycin\_biosynthetic\_gene\_cluster | 32 | 95.1492537313 | 111 | 1.6e-24 |
| CAA07765.1 | ketoreductase | BGC0000246 | Mithramycin\_biosynthetic\_gene\_cluster | 32 | 95.1492537313 | 111 | 1.6e-24 |
| CAA54861.1 | ketoreductase | BGC0000231 | Griseusin\_biosynthetic\_gene\_cluster | 32 | 95.8955223881 | 111 | 2.1e-24 |
| ANY57970.1 | Short\_chain\_dehydrogenase | BGC0001369 | Metatricycloene\_biosynthetic\_gene\_cluster | 30 | 96.2686567164 | 108 | 1.3e-23 |
| ACO31265.1 | Ptm01 | BGC0001140 | Platensimycin\_/\_platencin\_biosynthetic\_gene\_cluster | 32 | 95.5223880597 | 108 | 1.7e-23 |
| AEA30269.1 | putative\_3-oxoacyl-ACP\_reductase | BGC0000429 | Skyllamycin\_biosynthetic\_gene\_cluster | 30 | 95.5223880597 | 107 | 2.3e-23 |
| ADC45556.1 | 2-hydroxycyclohexanecarboxyl-CoA\_dehyrogenase | BGC0000093 | Meilingmycin\_biosynthetic\_gene\_cluster | 32 | 97.3880597015 | 106 | 3.9e-23 |
| EHM27508.1 | short-chain\_dehydrogenase/reductase\_SDR | BGC0000235 | Kiamycin\_biosynthetic\_gene\_cluster | 31 | 95.1492537313 | 106 | 5.1e-23 |
| CAA60572.1 | putative\_ketoreductase | BGC0000276\_c4 | Urdamycin\_biosynthetic\_gene\_cluster | 30 | 95.8955223881 | 106 | 5.1e-23 |
| ADD83002.1 | PtnO1 | BGC0001156 | Platencin\_biosynthetic\_gene\_cluster | 31 | 95.8955223881 | 104 | 1.5e-22 |
| AAM33669.1 | putative\_3-oxoacyl-ACP\_reductase\_GrhO2 | BGC0000230 | Griseorhodin\_biosynthetic\_gene\_cluster | 29 | 98.8805970149 | 104 | 1.9e-22 |
| AHD25940.1 | ChdT | BGC0000208 | Chelocardin\_biosynthetic\_gene\_cluster | 30 | 95.8955223881 | 103 | 3.3e-22 |
| AET51861.1 | 3-ketoacyl\_ACP\_reductase | BGC0001138 | A201A\_biosynthetic\_gene\_cluster | 29 | 93.6567164179 | 103 | 4.3e-22 |
| CBW45710.1 | putative\_ketoreductase | BGC0000952\_c3 | Pristinamycin\_biosynthetic\_gene\_cluster | 32 | 97.0149253731 | 102 | 9.6e-22 |
| AAN79649.1 | 3-oxoacyl-[acyl-carrier\_protein]\_reductase | BGC0000836 | APE\_Ec\_biosynthetic\_gene\_cluster | 30 | 93.6567164179 | 101 | 1.6e-21 |
| AAZ55906.1 | 2,3-dihydro-2,3-dihydroxybenzoate\_dehydrogenase;\_RBL00455 | BGC0000359 | Fuscachelin\_biosynthetic\_gene\_cluster | 32 | 97.3880597015 | 100 | 2.8e-21 |
| AEI70241.1 | 2,3-dihydro-2,3-dihydroxybenzoate\_dehydrogenase | BGC0000401 | Paenibactin\_biosynthetic\_gene\_cluster | 30 | 97.3880597015 | 99 | 4.7e-21 |
| ALG65315.1 | Cal21 | BGC0001297 | WS9326\_biosynthetic\_gene\_cluster | 28 | 102.23880597 | 98 | 1.4e-20 |
| ADE34504.1 | SsfK | BGC0000269 | SF2575\_biosynthetic\_gene\_cluster | 28 | 96.2686567164 | 97 | 2.4e-20 |
| AFO59869.1 | ChxH | BGC0000175 | Cycloheximide\_/\_actiphenol\_biosynthetic\_gene\_cluster | 31 | 101.119402985 | 96 | 6.9e-20 |
| BAC79042.1 | keto\_reductase\_(KR) | BGC0000245 | Medermycin\_biosynthetic\_gene\_cluster | 29 | 95.5223880597 | 95 | 1.2e-19 |
| CAA09651.1 |  | BGC0000227 | Granaticin\_biosynthetic\_gene\_cluster | 31 | 98.5074626866 | 94 | 1.5e-19 |
| CAA34367.1 |  | BGC0000228 | Granaticin\_biosynthetic\_gene\_cluster | 31 | 98.5074626866 | 94 | 1.5e-19 |
| CAA34368.1 |  | BGC0000228 | Granaticin\_biosynthetic\_gene\_cluster | 29 | 94.776119403 | 94 | 2e-19 |
| CAA09652.1 | polyketide\_ketoreductase | BGC0000227 | Granaticin\_biosynthetic\_gene\_cluster | 29 | 94.776119403 | 94 | 2e-19 |
| ABC42563.1 | putative\_short\_chain\_dehydrogenase | BGC0000698 | Hygromycin\_A\_biosynthetic\_gene\_cluster | 30 | 99.6268656716 | 92 | 5.8e-19 |
| CAB15190.2 | 2,3-dihydro-2,3-dihydroxybenzoate\_dehydrogenase | BGC0000309 | Bacillibactin\_biosynthetic\_gene\_cluster | 28 | 96.2686567164 | 92 | 5.8e-19 |
| AAM97360.1 | RubJ | BGC0000266 | Rubromycin\_biosynthetic\_gene\_cluster | 27 | 99.2537313433 | 92 | 7.6e-19 |
| ABS75236.1 | DhbA | BGC0001185 | Bacillibactin\_biosynthetic\_gene\_cluster | 29 | 96.6417910448 | 92 | 9.9e-19 |
| ABD65959.1 | acyl-CoA\_dehydrogenase/reductase | BGC0000341 | Enduracidin\_biosynthetic\_gene\_cluster | 27 | 102.23880597 | 91 | 1.7e-18 |
| ABI75134.1 | short-chain\_alcohol\_dehydrogenase | BGC0000887 | AWQC131C\_biosynthetic\_gene\_cluster | 32 | 70.8955223881 | 90 | 3.8e-18 |
| AAZ23052.1 | probable\_dehydrogenase | BGC0000291 | A54145\_biosynthetic\_gene\_cluster | 31 | 97.3880597015 | 89 | 4.9e-18 |
| ABI75108.1 | short-chain\_alcohol\_dehydrogenase | BGC0000928 | T3\_toxin\_biosynthetic\_gene\_cluster | 32 | 70.8955223881 | 89 | 6.4e-18 |
| BAR73014.1 | putative\_3-ketoacyl-ACP\_reductase | BGC0001194 | Hitachimycin\_biosynthetic\_gene\_cluster | 30 | 104.104477612 | 89 | 6.4e-18 |
| WP\_010369414.1 | 3-oxoacyl-ACP\_reductase | BGC0000314 | Bromoalterochromides\_biosynthetic\_gene\_cluster | 29 | 94.776119403 | 87 | 3.2e-17 |
| ERG36779.1 | 3-oxoacyl-[acyl-carrier-protein]\_reductase\_FabG | BGC0000299 | Alterochromides\_biosynthetic\_gene\_cluster | 29 | 94.776119403 | 87 | 3.2e-17 |
| ACU36662.1 | short-chain\_dehydrogenase/reductase\_SDR | BGC0000392 | Mirubactin\_biosynthetic\_gene\_cluster | 29 | 95.1492537313 | 85 | 9.3e-17 |
| CBA63665.1 | 2,3-dihydro-2,3-dihydroxybenzoate\_dehydrogenase | BGC0000368 | Griseobactin\_biosynthetic\_gene\_cluster | 29 | 98.8805970149 | 84 | 1.6e-16 |
| EDY47129.1 | conserved\_hypothetical\_protein | BGC0000845 | Clavulanic\_acid\_biosynthetic\_gene\_cluster | 28 | 70.8955223881 | 83 | 4.6e-16 |
| BAF98625.1 | putative\_2,3-dihydro-2,3-dihydroxy\_benzoate\_dehydrogenase | BGC0000651 | KS-505a\_biosynthetic\_gene\_cluster | 29 | 96.6417910448 | 82 | 1e-15 |
| BAK64641.1 | dihydroxy\_ketone\_synthase | BGC0000135 | Reveromycin\_biosynthetic\_gene\_cluster | 27 | 103.358208955 | 79 | 8.7e-15 |
| AAG31126.1 | MxcC | BGC0001345 | Myxochelin\_biosynthetic\_gene\_cluster | 27 | 95.8955223881 | 78 | 1.1e-14 |
| ADE22339.1 | beta-ketoacyl\_synthase | BGC0000065 | Galbonolides\_biosynthetic\_gene\_cluster | 27 | 88.4328358209 | 78 | 1.5e-14 |
| CBZ42141.1 | Acyl-Co\_Adehydrogenase | BGC0001117 | Himastatin\_biosynthetic\_gene\_cluster | 26 | 100.373134328 | 76 | 4.3e-14 |
| AAP11945.1 | Short\_chain\_dehydrogenase | BGC0000612 | Thiocillin\_I\_/\_thiocillin\_II\_biosynthetic\_gene\_cluster | 30 | 70.8955223881 | 75 | 9.6e-14 |
| BAO66523.1 | carveol\_dehydrogenase | BGC0000042 | Cremimycin\_biosynthetic\_gene\_cluster | 27 | 103.358208955 | 75 | 9.6e-14 |
| BAU98027.1 | dehydrogenase | BGC0001386 | Isofuranonaphthoquinone\_biosynthetic\_gene\_cluster | 27 | 75.3731343284 | 69 | 9e-12 |
| ACH85578.1 | CetF1 | BGC0000283 | Cetoniacytone\_A\_biosynthetic\_gene\_cluster | 27 | 101.865671642 | 68 | 1.2e-11 |
| CUW01207.1 | putative\_3-oxoacyl-ACP\_reductase | BGC0001359\_c2 | PM100117\_/\_PM100118\_biosynthetic\_gene\_cluster | 27 | 101.492537313 | 67 | 2.6e-11 |
| ABM63537.1 | BryA | BGC0000174 | Bryostatin\_biosynthetic\_gene\_cluster | 41 | 34.328358209 | 66 | 7.6e-11 |
| AAD37453.1 | putative\_ketoacyl\_reductase | BGC0000252 | Nonactin\_biosynthetic\_gene\_cluster | 26 | 87.6865671642 | 61 | 2.4e-09 |
| AAG34182.1 | SimK | BGC0000835 | Simocyclinone\_biosynthetic\_gene\_cluster | 28 | 67.1641791045 | 61 | 2.4e-09 |
| AAK06802.1 | putative\_3-keto-acyl-reductase\_SimD3 | BGC0001072 | Simocyclinone\_biosynthetic\_gene\_cluster | 28 | 67.1641791045 | 61 | 2.4e-09 |
| AAL15598.1 | SimK | BGC0000270 | Simocyclinone\_biosynthetic\_gene\_cluster | 28 | 67.1641791045 | 61 | 2.4e-09 |
| ADH01484.1 | putative\_type-I\_PKS | BGC0001096 | FR901464\_biosynthetic\_gene\_cluster | 30 | 70.5223880597 | 54 | 3e-07 |
| AIC32692.1 | FR9C | BGC0001113 | Spliceostatin\_/\_FR901464\_biosynthetic\_gene\_cluster | 30 | 70.5223880597 | 54 | 3e-07 |
| BAA89391.1 |  | BGC0000864 | Eicosapentaenoic\_acid-like\_compound\_biosynthetic\_gene\_cluster | 25 | 73.5074626866 | 52 | 1.1e-06 |
| ABI22118.1 | oxidoreductase | BGC0000422 | Saframycin\_A\_biosynthetic\_gene\_cluster | 33 | 36.9402985075 | 49 | 9.6e-06 |
